# Reciprocity With Unequal Payoffs: Cooperative and Uncooperative Interactions Affect Disadvantageous Inequity Aversion

**DOI:** 10.3389/fpsyg.2021.628425

**Published:** 2021-07-02

**Authors:** Carla Jordão Suarez, Marcelo Frota Benvenuti, Kalliu Carvalho Couto, José Oliveira Siqueira, Josele Abreu-Rodrigues, Karen M. Lionello-DeNolf, Ingunn Sandaker

**Affiliations:** ^1^Department of Experimental Psychology, Institute of Psychology, University of São Paulo, São Paulo, Brazil; ^2^National Institute of Science and Technology about Cognition, Behavior and Teaching, São Carlos, Brazil; ^3^Faculty of Health Sciences, Department of Behavioural Sciences, Oslo Metropolitan University, Oslo, Norway; ^4^Department of Basic Psychological Processes, University of Brasília, Brasília, Brazil; ^5^Department of Psychology, Assumption University, Worcester, MA, United States

**Keywords:** inequity aversion, reciprocity, cooperation, learning, points vs. money

## Abstract

Cooperation among unrelated individuals can evolve through reciprocity. Reciprocal cooperation is the process in which lasting social interactions provide the opportunity to learn about others' behavior, and to further predict the outcome of future encounters. Lasting social interactions may also decrease aversion to unequal distribution of gains – when individuals accept inequity payoffs knowing about the possibility of future encounters. Thus, reciprocal cooperation and aversion to inequity can be complementary phenomena. The present study investigated the effects of cooperative and uncooperative interactions on participants' aversion to disadvantageous inequity. Participants played an experimental task in the presence of a confederate who acted as a second participant. In reality, the participant interacted with a computer programed to make cooperative and uncooperative choices. After interacting with a cooperative or uncooperative computer, participants chose between blue cards to produce larger gains to the computer and smaller for him/her or green cards to produce equal and smaller gains for both. Results confirmed our first hypothesis that uncooperative interactions would produce aversion to disadvantageous inequity. Lastly, half of the participants were informed that points received during the experiment could be later exchanged for money, and half were not. Results indicated that information about monetary outcomes did not affect aversion to inequity, contradicting our second hypothesis. We discuss these results in the light of theories of reciprocal cooperation, inequity aversion, and conformity.

## Introduction

To explain the ways in which cooperation can evolve among unrelated individuals, Trives (1971) proposed the notion of reciprocity. Trives defined reciprocity as one individual providing helpful acts toward another individual who can provide benefits in return at a later time. Nowak and Highfield (2012) presented several theoretical models in which cooperation can evolve when reciprocity occurs (for review, see Nowak, 2006 and Rand and Nowak, 2013). Szolnoki and Perc (2015) discussed how conformity (i.e., choosing the most common strategy within a group) can increase reciprocity in social dilemmas and consequently affect cooperation. Also, several behavioral experiments have shown characteristics and variables that affect reciprocity. For example, children and adults cooperate more when playing with the same partner multiple times (e.g., Dal Bó, 2005; Dal Bó and Fréchette, 2011; Blake et al., 2015b). Children exhibited higher degrees of reciprocity when exposed to cooperative puppets in a pretest condition (Vaish et al., 2018). Adults made larger donations after interacting with cooperative confederates (Smith et al., 2017). Children and adolescents used cooperative strategies when playing with a cooperative confederate (Keil et al., 2017). Adults' reciprocal responses in a puzzle game were modulated by confederates' percentage of cooperative plays (e.g., Ribes-Iñesta et al., 2010; Avalos et al., 2015). In a prisoner's dilemma game, adults cooperated when the probability of reciprocity was high between the dyad (e.g., Silverstein et al., 1998; Baker and Rachlin, 2001).

A key aspect in cooperation by reciprocity is the probability of future interactions (Axelrod and Hamilton, 1981; Axelrod, 1984). In this sense, experimental studies on reciprocity using within-subjects designs may be useful in exploring how such mechanisms interact with learning principles and produce cumulative changes in an individual's cooperative behavior (Rachlin, 2019). Studies that investigate how reciprocity may change due to learned mechanisms are important for understanding the variability and idiosyncrasies of participants' choices and their contextual modulation. Thus, reciprocity strategies may be also interpreted from the perspective of basic principles of learning considering the ways in which socially mediated reinforcement influences past, present, and future reciprocal behavior (Schmitt, 1998; Baker and Rachlin, 2001; Rachlin et al., 2001).

Payoffs distributions between participants may vary in most social exchanges. Sometimes, unequal distribution may be experienced as aversive (Oberliessen and Kalenscher, 2019). In fact, behavioral studies about unequal payoffs with non-humans (Brosnan and de Waal, 2003), babies (Schmidt and Sommerville, 2011), children (LoBue et al., 2011), adolescents (Blake et al., 2014, 2015a), and adults (Bone and Raihani, 2015) have shown that humans and non-humans may refuse to accept unequal distributions of gain. Ferh and Schmidt (1999) and Brosnan (2011) suggested that aversion to inequity and cooperation are intertwined processes, in which individuals discriminate when a partner received unfairly benefits from social exchanges and uses such information for future interactions. Consistent with this, Perc (2000) and Perc and Szolnoki (2008) observed that the distribution of wealth (i.e., distribution of unequal outcomes) plays a crucial role in the evolution of cooperation among unrelated individuals. Also, Hauser et al. (2019) argued that extreme payoff inequity prevents cooperation. However, if individuals differ in productivity, inequality may be necessary and tolerated for cooperation to prevail. Individuals who cooperate for extended periods of time may manipulate payoff distributions, further effecting inequity either toward or away from a fair distribution. Perc (2000), Perc and Szolnoki (2008), and Hauser et al. (2019) are consistent with the proposition that unequal distributions are not always seen as unfair (Starmans et al., 2017). Therefore, aversion to inequity should be studied as another evolutionary process that occurs at the group level and over extended periods of time (i.e., altruism; Wilson and Wilson, 2007).

Two types of inequity aversion have been described in the literature: aversion to advantageous inequity (AI) and aversion to disadvantageous inequity (DI). The latter refers to aversion to unequal and unfavorable payoffs, and the former refers to aversion to unequal and favorable payoffs (e.g., Blake and McAuliffe, 2011; McAuliffe et al., 2013, 2017). Studies of aversion to inequity often employ an unequal distribution of outcomes to perform a common task and examine an individual's refusal to accept unequal gains as the dependent variable (Ferh and Schmidt, 1999; Ahmed and Karlapalem, 2014; Brosnan and de Waal, 2014; Blake et al., 2015a). Overall, studies of inequity aversion have used tangible rewards as dependent variables for both humans and non-human participants. Such studies may use candy or stickers to study aversion to inequity in children (Blake and McAuliffe, 2011; LoBue et al., 2011; Shaw and Olson, 2013; Blake et al., 2014; Corbit et al., 2017), money in studies with adults (Schmitt and Marwell, 1971a,b; Schmitt and Marwell, 1972; Shimoff and Matthews, 1975), and cucumbers and grapes in studies with monkeys (Brosnan and de Waal, 2003). Blake and Rand (2010) showed that reward value affects children's donations to other children. The amount donated by the children was inversely proportional to the value of the reward for them; that is, children preferentially donated their non-preferred stickers and rarely donated their favorite stickers. In the present study, one of our aims was to investigate the role of the nature of outcomes over inequity aversion (inform participants or not that earned points were exchangeable for money) in a between-subject design.

Aversion to inequity may be affected by the nature of outcomes. However, cooperative interactions also modulate whether individuals perceive inequal distribution as aversive (e.g., Silverstein et al., 1998; Avalos et al., 2015; Corbit et al., 2017; Benvenuti et al., 2020). Corbit et al. (2017), for example, investigated the effects of a prior cooperative interaction on inequity aversion in dyads of children in India and Canada. In the pretest condition, the dyads worked on a cooperative task, in which both children had to simultaneously pull a rope to move a container with candies or stickers, or on an individual task, in which each child individually pulled the rope. In the test condition, the participants played an Inequity Game that evaluated their aversion to AI and DI. The children sat opposite each other, and the obtained rewards were individually or cooperatively distributed by the experimenter. One child in each dyad was selected to choose a green lever to accept the distribution or a red lever to refuse it. If the distribution was refused, then neither child in the dyad would receive the rewards. The dyads were randomly distributed into four groups: Cooperative Task and AI, Cooperative Task and DI, Individual Task and AI, and Individual Task and DI. Children from both India and Canada exhibited aversion to AI after being exposed to the cooperative task but not to the individual task. The children refused disadvantageous distribution regardless of the training task (i.e., cooperative or individual). These and other results suggest that aversion to AI can be modulated by social interactions and cultural context, whereas DI is less sensitive to this (e.g., Adams, 1965; Ferh and Schmidt, 1999; Takagishi et al., 2010; Blake and McAuliffe, 2011; Blake et al., 2014, 2015a; McAuliffe et al., 2017; Li et al., 2018). The results produced by Corbit et al. (2017) suggest that cooperation modulates AI aversion but has no effect on DI aversion. As reciprocity is known to have a strong effect on participants' choices to cooperate (e.g., Schmid et al., 2021), we investigated whether cooperative and uncooperative interactions involving reciprocity would affect aversion to DI. The elucidation of the connection between reciprocity and aversion to DI may represent a novel contribution to the understanding of the evolution of cooperation at large.

In the present study, we explicitly investigated how cooperation by reciprocity affects college students' decisions to accept or refuse unequal disadvantageous gains. We examined whether cooperative and uncooperative interaction between two individuals would influence participants' willingness to produce or refuse disadvantageous inequity. To do so, we arranged experimental conditions in which the “other participant” (i.e., programed computer) worked cooperatively or uncooperatively in a situation that potentially produced greater gains for the participant (i.e., advantageous inequity for the participant). Next, participants were exposed to a test condition, where the computer could maximize gains if the participant behaved cooperatively or earn fewer points if the participant did not cooperate. Participants uncooperative responses in test conditions were defined as disadvantageous inequity aversion, that is, participants refused unequal and disadvantageous gains. We aimed at investigating whether (i) interactions with a cooperative computer would affect reciprocal cooperation in test conditions and whether (ii) interactions with an uncooperative computer would produce disadvantageous inequity. This experimental procedure was implemented with participants who were informed that points would be exchanged for money by the end of the experiment and participants who were not informed. Information about money exchange was used to evaluate a third research question, whether (iii) the value of reinforcers for cooperating influenced reciprocal cooperation and aversion to inequity.

## Materials and Methods

### Participants

Forty-three university students (26 women and 17 men), 18–29 years of age, participated in the experiment. The participants were undergraduate students in the following majors: civil and electrical engineering, physics, psychology, and administration. Although participants were asked not to talk about the experiment, we had no control over whether they followed the instruction or whether they knew each other. All of the participants signed an informed consent form that was approved by the ethics committee of the University of São Paulo. Experimental procedures, including instructions given to participants, were also approved by the ethics committee (CAAE protocol: 64336716.4.0000.5561). The participants were informed that they would perform the task as part of a dyad, but the choices were actually made by a computer program. To simulate the participation of the other participant, a confederate remained in the experimental room throughout data collection, making some noises, such as dragging the chair and writing on paper when necessary.

### Settings and Materials

**Figure 1:**
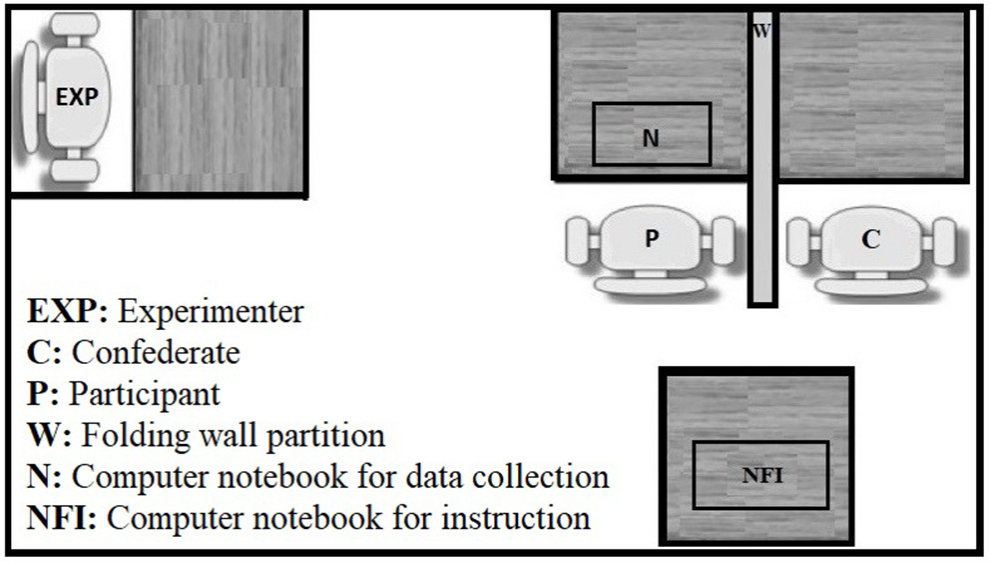
An overhead depiction of the experimental setting, including the table and chairs disposition in the experimental room, as well as the position where the confederate, the participant and the experimenter remained throughout the experiment.

Experimental sessions occurred at the University of São Paulo, São Paulo, Brazil. The sessions were conducted in a room (25 m^2^; Figure 1) that contained four desks, three chairs, a folding wall partition, two computer notebook computers (Samsung FR511, Intel i7-2670QM, Windows 7 Professional 64-bit operating system), a mouse, and a headset. When the participant entered the room, the confederate was already seated in one of the chairs, as shown in Figure 1. The participant and confederate were seated side-by-side, separated by the wall partition such that eye contact between them was not possible. One computer was placed on the table in front of the participant. The other computer was placed on a table located directly behind the participant and confederate. In order to mask extraneous room noise, a white sound was played on the participant's headset. The Interdependent Response and Consequence Programming software (ProgRCI) displayed the task, recorded the participant's responses, and simulated the other participant's choice, which will be described along with the paper as *computer*'s choices.

### Experimental Task

**Figure 2:**
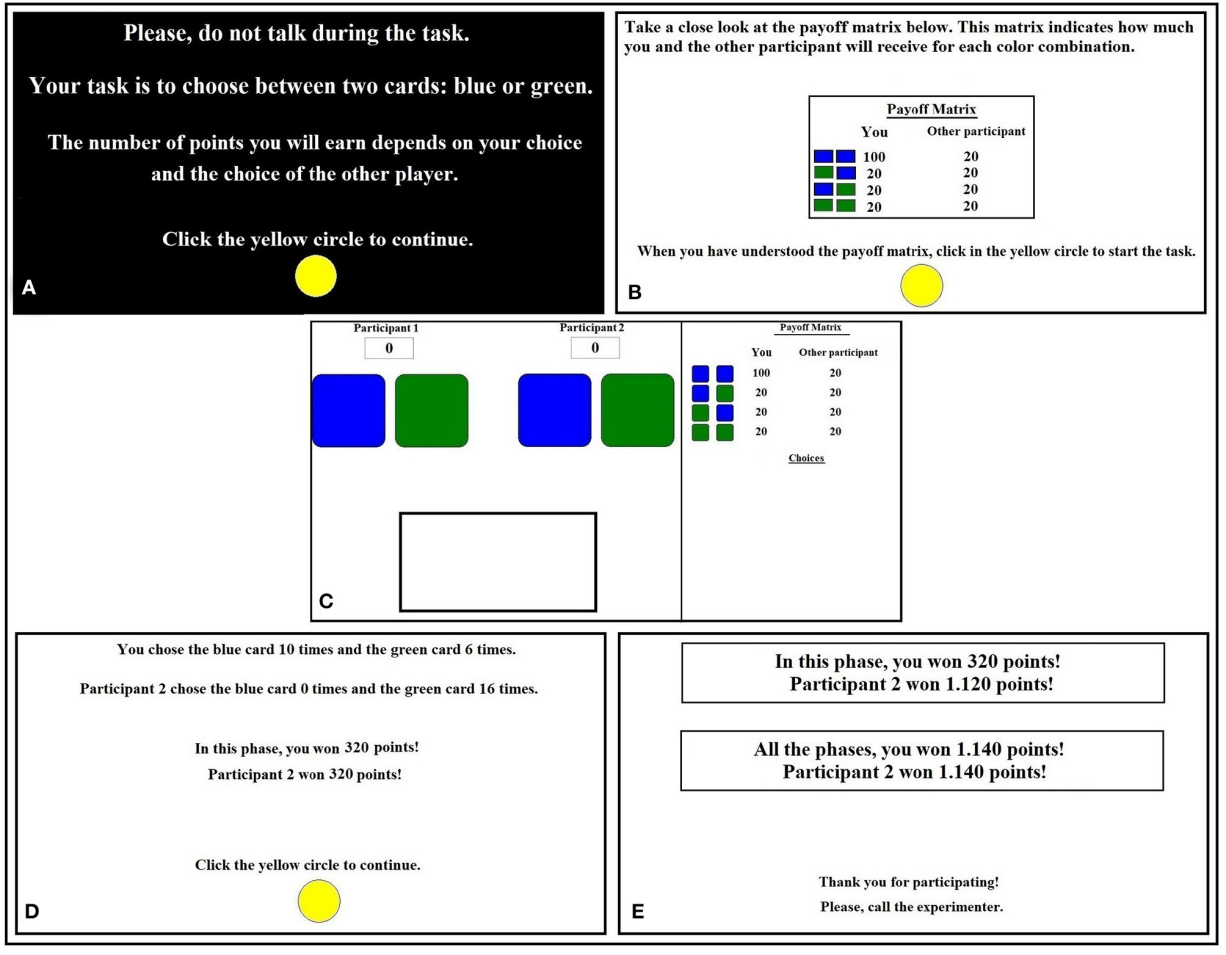
Screens shown during the experimental session. **(A)** Presented a summary of the instructions. **(B)** Presented the payoff matrix. Only the payoff matrix used in the cooperative interaction and uncooperative interaction condition is presented. **(C)** Presented the experimental task. **(D)** Presented a matrix of points corresponding to the new condition. **(E)** Presented a final screen showed the number of points gained in the previous condition as well as the total number of points obtained in the experiment.

The experimental task of this study was similar to that presented by Benvenuti et al. (2020). In each trial, two sets of two virtual cards (blue and green) were displayed on the participant's computer screen. One set represented the participants' cards, and the other set represented the computer's cards. In each trial, the participants were asked to choose between a green or blue card. Figure 2 shows all screens that were displayed during the experimental session.

The first screen presented a summary of the instructions (Figure 2A). The participants were asked to click on the yellow circle to move to the next screen. The second screen showed the matrix of points that were in effect for the current experimental condition (Figure 2B). Thereafter, the participants were instructed to press the yellow button again to start the task. The task screen (Figure 2C) showed the virtual cards and payoff matrix, which depicted the point outcomes for each card combination for that trial. After the participants clicked on one of the chosen cards (i.e., blue or green), the points that were gained during the trials accumulated in a point counter that was located at the top left of the screen and the color combination of each trial displayed the card chosen by the participant and the computer program (Figure 2C). At the end of each trial was a 1-s intertrial interval, during which the screen became entirely white. The computer cursor was placed back in the middle of the screen at the start of each trial, ensuring that the participants actively made a new choice in every trial. At the end of each condition, a screen that showed the choices that were made by the participants and computer program and points that were received were displayed (Figure 2D). Then, a screen with a matrix of points corresponding to the new condition was presented and followed by the screens described above. At the end of the session, a final screen showed the number of points gained in the previous condition as well as the total number of points obtained in the experiment. It also asked the participant to call the experimenter (Figure 2E).

### Procedure

When entering the room, the participant was greeted by the experimenter and invited to sit in one of two chairs. By that time, the confederate was waiting in the experimental room, sitting in the second chair. From their chair, the participant had visual access to the notebook to be used to perform the task, located in front of his/her chair and to the notebook for instructions (NFI) positioned at his/her back (Figure 1). A simulation that used the NFI explained the payoff matrix before beginning the experimental session. The experimenter first informed the participant that he/she would be Participant 1 throughout the experiment and that the “other participant” (i.e., computer program) would be Participant 2. The experimenter showed one example of a matrix of points on the screen of the NFI and orally explained how to perform the experimental task (i.e., choose one of the cards and click on it). The experimenter also emphasized that the number of points that were received would depend on the color combination that is produced by Participant 1 and Participant 2. A simulation was then performed to verify that the task and the matrix were understood by the participant.

#### Simulation Trials

Four trials with alternating different types of choices were simulated, such that all color combinations were presented. At the end of each simulated trial, the experimenter asked the participant to write on paper how many points they would receive for each color combination. For example, in the first trial, the experimenter said, “Let us assume that Participant 1 chose the blue card and Participant 2 chose the green card. With this color combination, how many points would each of you receive? Please do not speak out loud. Write your answer on the paper and I will go to your desk to check it out.” If the participant's answer was incorrect in any of the simulation trials, then the experimenter would explain the matrix of points again and moved on to another type of trial.

#### Instruction

After the simulated trials, the experimenter gave the written general instructions and asked to read it silently. The instructions were the following (translated from Portuguese):

“Hello Participant. Thank you for participating in this research project! This study is not about intelligence or emotions. You will be working with a partner, and both of you will have an identical task to perform during the experiment. You and your partner must choose between two cards (one blue and one green). In each trial, you will receive a certain number of points. The number of points you will receive depends on your choice and your partner's choice. The matrix of points that will appear to you is the same as the one that will appear to the other participant. Please remain seated and do not talk to your partner or experimenter during the session. If you have any questions or if you need to ask a question, then raise one hand, and the experimenter will come to you. Once the experiment is over, the following message will appear: “Thank you very much for participating! Please call the experimenter!” When this message appears, raise one of your hands, and the experimenter will come to you. Put on the headset that is lying on your desk and start the task.”

After handing over the paper with instructions to the participant and the confederate, the experimenter asked them to put on the headset and start the experimental session. At the end of the session, the experimenter asked the participant and the confederate to complete a questionnaire with two questions: The first question was: Why did you choose the blue or green card? You can check more than one alternative if necessary. To answer this question, the participant and the confederate had to choose one of the following alternatives: (a) To receive more points than the other participant; (b) It depended on the choice of the other participant; (c) To keep the same score between the two participants; (d) None of the previous alternatives (please, explain your answer). The second question was: In your opinion, why did the other participant choose the blue or green card? You can check more than one alternative if necessary. The alternatives were: (a) To receive more points than me; (b) It depended on my choice; (c) To keep the score equal between you and him/her; (d) None of the previous alternatives (please, explain your answer). If the participant chose the letter (d) for any of the questions and wrote that the other player was not a real participant but the computer, his/her data were not analyzed. If the participant did not explain why he/she chose the letter d, the experimenter asked what he/she thought and wrote it down on the paper. None of the participants who met the criteria to participate in the experiment chose letter “d” in the post-experimental questionnaire.

### Experimental Conditions

All participants were exposed to three experimental conditions: equity, cooperative interaction, or uncooperative interaction, and disadvantageous inequity test. There were three payoff matrices that differed in terms of the distribution of points between the participant and the computer when the blue-blue combination occurred (see Table 1). Each condition consisted of 16 trials.

**Table 1:** Points received by the participant and the computer with each card color combination and the number of trials in each condition.

**Condition**	**Color combination**		**Points**		**Number of trials**
			**Participant**	**Computer**	
Equity	Blue	Blue	100	100	16
	Any other		20	20	
Cooperative interaction	Blue	Blue	100	20	
Uncooperative interaction	Any other		20	20	
Disadvantageous Inequity	Blue	Blue	20	100	
	Any other		20	20	

#### Equity

The blue-blue color combination resulted in 100 points for the participant and the computer. Any other combination resulted in 20 points for both. The computer was programmed to choose the blue card 12 times and to choose the green card four times at random. In this condition, the participant and the computer always received the same number of points.

#### Cooperative Interaction (CI)

The blue-blue combination resulted in 100 points for the participant and 20 points for the computer. The remaining possible combinations resulted in 20 points for the participant and 20 points for the computer. In this condition, the computer was programmed to choose only the blue card. With that, participants were able to receive 100 by choosing the blue card, in which case the computer received 20 points. This computer program was considered cooperative as choices favored participants earns.

#### Uncooperative Interaction (UI)

As with the condition described above, the participant received 100 points with the blue-blue combination. However, the computer was programmed to choose the green card on all trials. Thus, regardless of the participant's choice, equity in the distribution of points was the only possible outcome. The computer choice did not allow participants to earn 100 points on any trial, thus defined as uncooperative.

#### Disadvantageous Inequity Test (DI – Test)

The blue-blue combination resulted in 20 points for the participant and 100 points for the computer. The other three possible combinations resulted in 20 points for the participant and 20 points for the computer. The computer was programmed to choose the blue card on every trial. This condition followed both CI and UI. Participants choice of green card did not allow the computer to receive 100 points, and thus defined aversion to inequity.

All participants were exposed to the four conditions described above; however, they were assigned to one of two sets of experimental instructions. The instructions for half of the participants did not mention that points earned during the experimental task would be later exchangeable for money. The instructions for the other half of the participants had an additional sentence stating that points earned during the experiment would be later exchanged for money. The sentence was written as follows (translated from Portuguese): “The points you receive will be exchanged for money at the end of the experimental session.” Regardless of how many points the participants earned in the experiment, everyone received R$10 (~USD$2.50) to reimburse transportation costs.

### Experimental Design

Participants were exposed to a mixed design (Johnston and Pennypacker, 2009) in which one of the independent variables – cooperative or uncooperative interaction – was manipulated within-subjects, and the other independent variable – payoff nature (i.e., points and money) – was manipulated between subjects.

**Figure 3:**
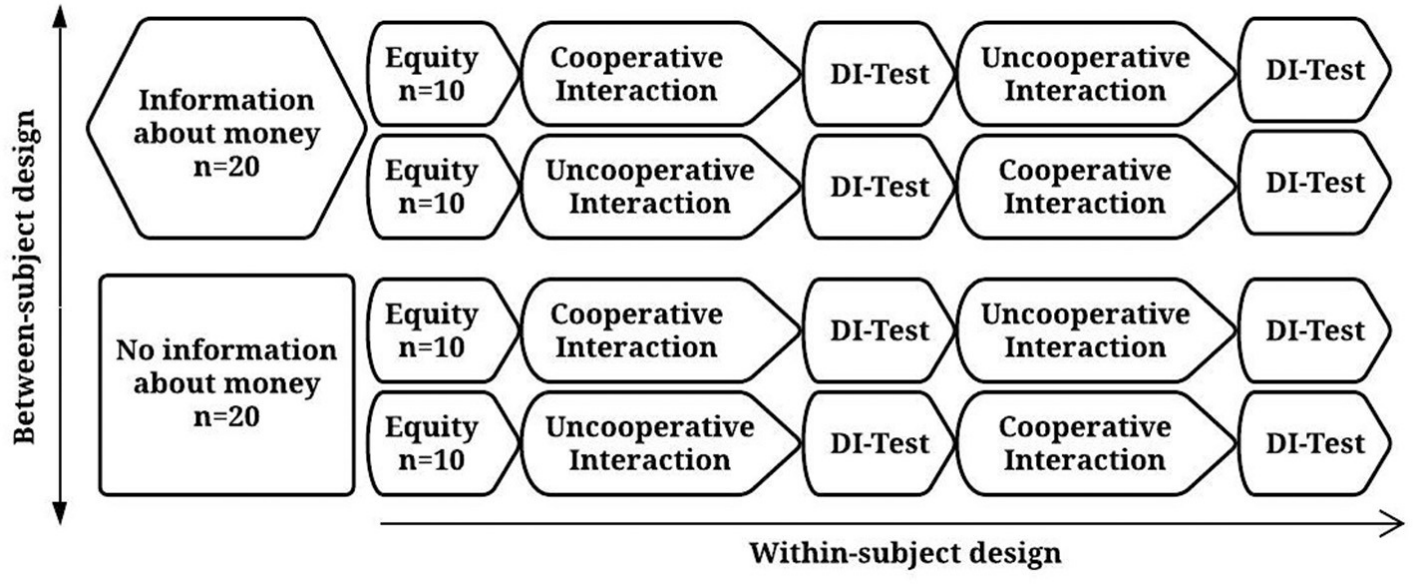
Flowchart of experimental design and conditions order of exposure. Experimental design consisted of a within group design, in which half of the participants (*n* = 20) received information that points would be exchanged for money and the other half (*n* = 20) did not (left hexagon and rectangle), and within-subject design as participants from both groups were exposed to cooperative and uncooperative interaction in different orders.

All recruited participants (*n* = 43) were exposed initially to the equity condition such that their choices could be observed in a situation in which all card combinations produced equal outcomes, but a blue-blue combination produced a better outcome for both. If the participant did not choose the blue card in at least 12 of 16 trials, his/her results were not included in the data analysis. In total, 40 participants met this criterion. We assessed possible effects of the order of the experimental conditions, so 20 participants were exposed to the following order of experimental conditions: Cooperative Interaction (CI), Disadvantageous Inequity Test (DI-Test), Uncooperative Interaction (UI), and lastly, Disadvantageous Inequity Test (DI-Test). The other 20 participants reverse order: UI, DI-Test, CI, and DI-Test. The rationale for this design was to observe participants' choices in DI-Test conditions after interactions in which the computer was programmed to be cooperative or uncooperative with the participant. Figure 3 represents a flowchart of experimental design and conditions order of exposure.

As we show in Figure 3, after receiving or not information that points would be exchanged for money (left hexagon and rectangle), participants responded to the equity condition (center circles). Thereafter, participants responded to one of the sequences of the conditions (rectangle on the right).

The experimental design illustrated in Figure 3 was planned to investigate whether (1) a history of cooperative or uncooperative interaction would affect aversion to inequity differently, whether (2) this influence depends on the order in which UI and CI conditions were presented, and whether (3) aversion to disadvantageous inequity is modulated by the information that points would be later exchanged for money. Our hypotheses were:

**Hypothesis 1**. Several studies (e.g., Corbit et al., 2017; McAuliffe et al., 2017) have suggested that DI aversion is less sensitive to social interactions and cultural context than AI aversion. On the other hand, the literature on cooperation shows that it is strongly affected by reciprocal interactions (Silverstein et al., 1998; Baker and Rachlin, 2001). The main hypothesis of the present study is that participants' choices during a DI-Test will be affected by reciprocal cooperation. That is, CI will decrease, and UI will increase, aversion to DI during a subsequent DI-Test condition. We tested this hypothesis by manipulating CI and UI interactions and testing DI aversion in a within-subjects design (see Figure 3).
**Hypothesis 2**. A diversity of rewards (tangible and non-tangibles) has been used in studies investigating the connection between DI and cooperation in non-humans, adults, and children (e.g., Blake and Rand, 2010; Blake et al., 2014; Brosnan and de Waal, 2014). We tested whether or not receiving information that earned points would be exchanged for money affected levels of aversion to inequity in a DI-Test in a between-subjects design (see Figure 3). In this hypothesis, we predicted that participants would show higher levels of aversion to inequity when receiving information about the points' monetary value.

### Data Analysis

During DI-Test conditions, Participants choices of blue cards allowed the computer to earn more points, and choices of green card prevented the computer from earning more points. Thus, the proportion of blue-card choices was used as a measure of DI aversion. A within-subjects design was employed to evaluate participants' proportion of blue-card choices after interacting with a cooperative and uncooperative computer (hypothesis 1). The effects of receiving information about the nature of outcomes (money or points) was evaluated in a between-subjects design (hypothesis 2). We used IBM SPSS Statistics 26 to perform a Generalized Linear Mixed Model (GLMM) with procedure GENLINMIXED. The dependent variable is the dichotomous nominal variable of the participants' choice of a blue or green card, with green as the reference category. We used the binomial distribution and logistics link function resulting in a repeated measure binary logistic regression model. The participant identification variable was modeled as a random effect. The fixed main effects are the card choices by the computer programming during cooperative and uncooperative conditions and the participant's choice of cards in DI-Test conditions. The fixed interaction effect is composed of the programming variables of the computer card choice and the conditions of the experiment (order of interactions and information about monetary outcome). The significance level used in each null hypothesis test was 5% (see Appendices A and B for raw data).

## Results

**Figure 4:**
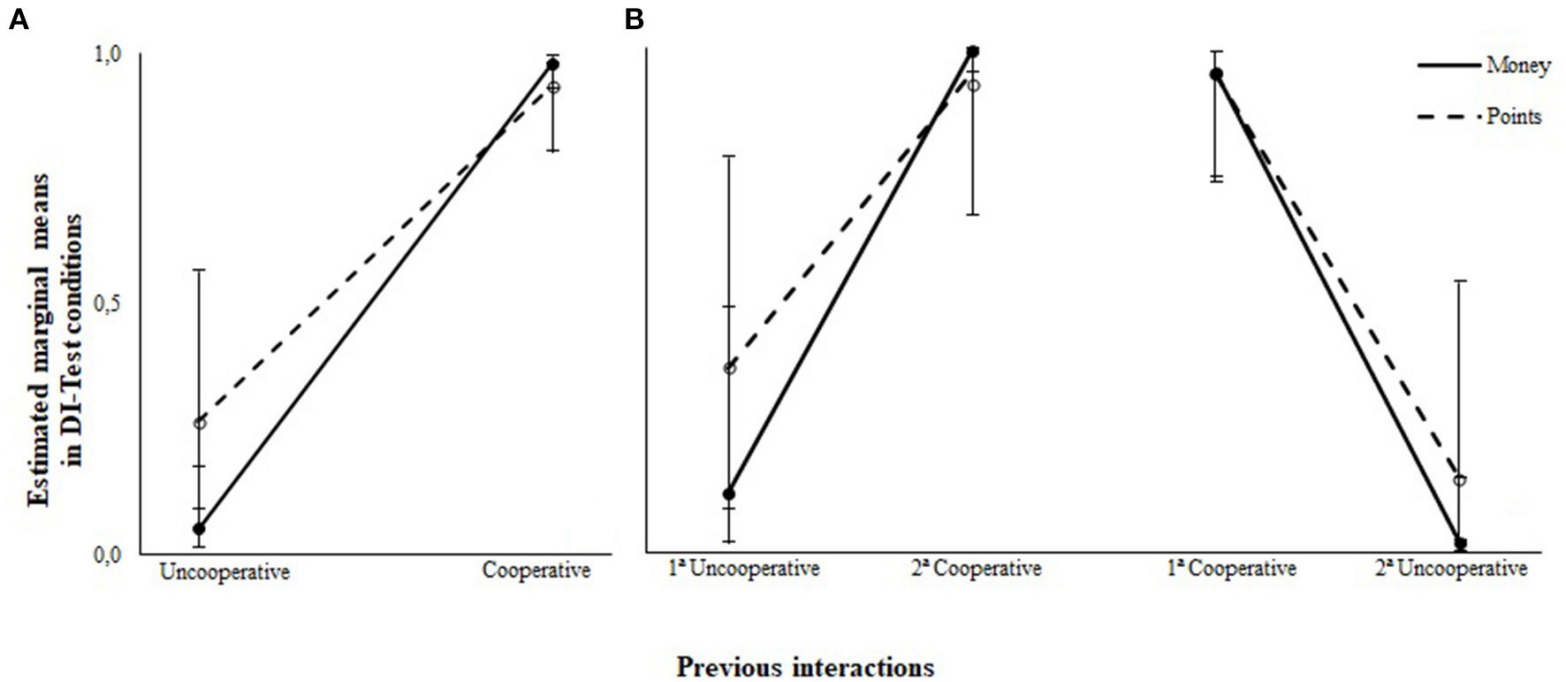
Estimated marginal means of participant's blue cards choices under disadvantageous inequity test conditions as a function of previews interactions with cooperative and uncooperative computer. **(A)** Depicts participants aggregated data for proportion of blue choices, showing that computer choices prior to DI-Test directly affected participants' choices. **(B)** Depicts participants proportion of choices of blue card in each DI-Test, showing that prior interactions directly affected participants' choices in DI-Test conditions regardless of the order of exposition.

The present study evaluated participants' choices in the DI-Test conditions after interactions with a cooperative and uncooperative computer. A GLMM omnibus test was used to assess hypothesis 1. The results from the fixed-effect test accounting for the variables information about monetary outcomes and DI-Test conditions showed a significant difference (*F*_(3,1272)_ = 8.203, *p* < 0.001) between proportion of blue-card choices after interaction with a cooperative and uncooperative computer. This result confirms hypothesis 1 – participants showed higher proportions of reciprocity after cooperative interactions. Figure 4A depicts the proportion of blue-card choices in the DI-Tests after cooperative and uncooperative interactions separately for the participants who received information about money and those who did not. This figure shows that choices of blue cards were substantially higher in the DI-Test following the CI interaction than the UI interaction.

The within-subjects design employed to evaluate hypothesis 1 allowed testing to determine whether the order of presentation of cooperative and uncooperative interactions differentially affected DI aversion. The *post-hoc* analysis revealed no significant difference between the proportion of blue-card choices when accounting for order of presentation of cooperative and uncooperative interactions. Regardless of whether the participants were informed about monetary outcomes, there was an overlap of <25% in the confidence intervals, with *t*(1272) = −8.74, adjusted *p* < 0.001 and *t*_(1, 272)_ = −3.50, adjusted *p* = 0.002. This *post-hoc* pairwise analysis revealed reciprocal cooperation after interacting with a cooperative computer and aversion to DI after interacting with an uncooperative computer was not affected by presentation order. Figure 4B shows the estimated marginal means for proportion blue-card choices in DI-Test conditions for participants exposed first in the uncooperative interaction and then in the cooperative interaction, and for the participants exposed first to the cooperative interaction and then to the uncooperative interaction at corresponding 95% confidence intervals.

**Figure 5:**
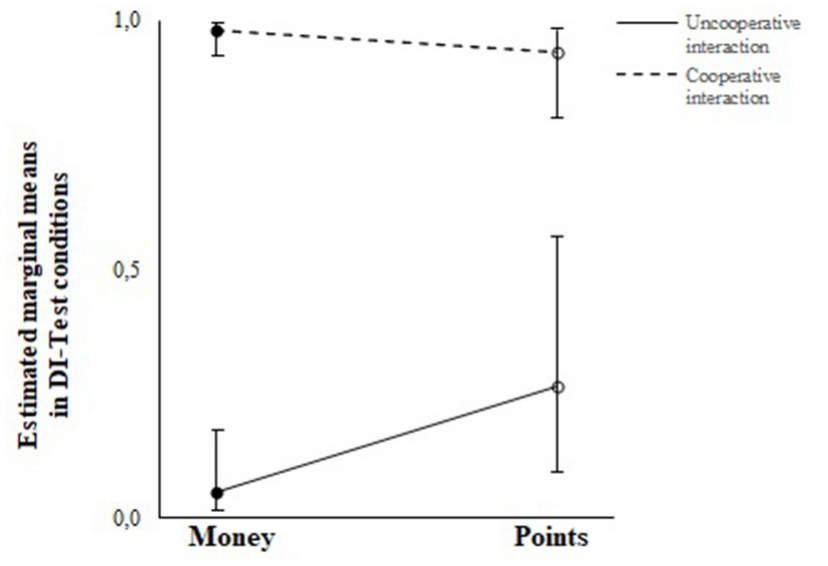
Estimated marginal means of participant's blue cards choices under disadvantageous inequity test conditions as a function of the information about monetary outcomes.

A second *post-hoc* analysis was carried out to evaluate hypothesis 2 – whether receiving information on the monetary outcomes would affect participants' reciprocity under DI-Test conditions. The results of these *post-hoc* tests revelead a significant effect of cooperative and uncooperative interaction on reciprocity, when not considering information about monetary outcome (money group: *F*_(3,1272)_ = 983, *p* < 0.001; points group: *F*_(3,1272)_ = 33, *p* < 0.001). However, the *post-hoc* analysis showed no statistically significant difference in the proportion of blue-card choices when considering information about monetary outcome (*F*_(1,1272)_ = 1.029, *p* = 0.295). Thus, the conclusion is that the monetary outcomes within each DI-Test condition had no significant main effect on reciprocity. Figure 5 shows the estimated marginal mean proportion of blue-card choices after cooperative and uncooperative interactions for participants who did and did not receive information about monetary outcome.

## Discussion

The results of the present study support the main hypotheses (hypothesis 1): Despite disadvantageous inequity, the participants cooperated under DI-Test conditions in a similar proportion that the computer was programmed to cooperate under prior conditions. That is, choices in the DI-Test followed a reciprocal cooperation strategy: After interacting with a cooperative computer (CI), the participants were also cooperative, and after interacting with an uncooperative computer (UI), the participants were also not cooperative. This reciprocal cooperation strategy was observed regardless of conditions CI and UI order of presentation. As far as we know, this is the first study that clearly shows how reciprocity can alter aversion to disadvantageous inequity in a within-subject design. Our data suggest that the same participant may be or may be not averse to disadvantageous inequity due to recent past cooperative history.

These data support the hypothesis that by interacting multiple times, participants have the possibility to learn about their partner's choices and modulate their behavior accordingly in future interactions, as suggested by Baker and Rachlin (2001). Thus, a well-controlled experimental history of social interaction that involves different magnitudes of gains (i.e., more or fewer points for the participant in CI and UI interactions conditions affected the participants' choices under subsequent conditions, a result that is consistent with the literature on the effects of a history of reinforcement (and punishment) on non-social (e.g., Sidman, 1960; Galizio, 1979; Freeman and Lattal, 1992; Okouchi, 1999) and social (e.g., Mithaug, 1969; Buskit and Morgan, 1987; Spiga et al., 1992; Abreu-Rodrigues et al., 2002) situations. Results replicated findings in children (e.g., Keil et al., 2017; Smith et al., 2017; Vaish et al., 2018) and adults (e.g., Ribes-Iñesta et al., 2010; Avalos et al., 2015), indicating that participants are more cooperative in a test condition after being previously exposed to cooperation. Moreover, results suggest that under the present experimental conditions, the interaction of successful cooperation between two individuals may cumulatively increase the likelihood that a particular individual will act in a way that produces DI for himself/herself. These data may be consistent with the general proposition of Tomasello et al. (2005) about the ability of humans to share intentions in cooperative tasks. The probability of future interactions and their role in cooperation can be seen as a byproduct of experience in which learning about another's intention to be cooperative or uncooperative.

We also asked whether information about the value of the outcomes (points alone or points exchangeable for money) would affect levels of inequity aversion during DI-Test conditions (hypothesis 2). Our data show no significant difference between aversion to inequity and information about experiment outcome. These results contradict studies that have investigated whether the nature of earnings (points, money, candy, toys) affected participants' choices (Harbaugh et al., 2001; Blake and Rand, 2010; Salgado et al., 2011). One possible explanation for the inconsistency across studies is that the influence of the history of successful or unsuccessful cooperation in CI and UI exceeded the possible influence of points vs. monetary gains. One alternative way to investigate differences between points vs. monetary gains would be to expose participants directly to disadvantageous inequity without prior experiences. This manipulation would isolate the effects of points/money from those of cooperative or non-cooperative interaction and may reveal differential effects of those outcomes.

Results are also consistent with assertions by Brosnan (2011) about the relationship between cooperation and inequity aversion. Her hypothesis states that inequity aversion allows individuals to evaluate when they should (a) discontinue interactions with partners who continually accept advantageous inequity and, thus, benefit more in cooperative situations, (b) replace such unfair partners with partners who prefer equal distribution of gains, and (c) encourage future interactions with equitable partners and/or manage their reputation as a fair partner by refusing advantageous inequity. For Brosnan, such strategies can play an important role in long-term cooperative actions, particularly when unrelated individuals benefit from reciprocity and mutualism. Importantly, our results show that interactions can modulate shifts in aversion to disadvantageous inequity and reciprocal cooperation within the length of an experimental session.

Several experimental studies of inequity aversion showed that non-human primates (Brosnan and de Waal, 2003), babies (Schmidt and Sommerville, 2011), very young children (LoBue et al., 2011), and adolescents and adults (Blake et al., 2014, 2015a; Corbit et al., 2017) refused disadvantageous distributions. In the study by Corbit et al. (2017), for example, children refused to receive more than others after a cooperative interaction. In contrast to the present results, however, they also refused disadvantageous distributions of gains. Such differences may be attributable to the type of task and specific experimental design. In the present study, we could specify the reciprocal relationship that was established between the participants. In Corbit et al.'s (2017) study, different experimental tasks were used (a task prior to the situation of inequity and the Inequity Game). In the present study, with the same experimental task across conditions, it was possible to evaluate whether the participants' choices were reciprocal to the confederate's choices or not. Thus, our results highlight the importance of considering reciprocal cooperation and aversion to inequity as complementary phenomena.

Results can also be analyzed through a theory of conformity. Although individuals seek to maximize earnings in social interactions, choosing the most used strategy (i.e., conformity) can guarantee minimal payoffs at group average levels. In addition, the acceptance of the group in relation to one's choice can function as a social reinforcer for conformity, and a sense of belonging (Szolnoki and Perc, 2015). Szolnoki and Perc (2015) argue that individuals may conform to others in order to select the most promising strategies for future interaction within the group. Regarding the results presented here, conformity may have played a role in the influence of computer cooperative strategies during CI and UI over participant's choices in DI-Test. Future research may study the relationship between aversion to inequity and conformity by designing experimental tasks where more than two participants exert influence over each other, and as part of a group.

Research on reciprocity has created interesting discussions about the evolution of cooperation and has contributed to the design of quantitative models of social behavior and cultural evolution (Boyd and Richerson, 1985). The learning experience may interact with all of these factors. Investigations of interactions between them may lead to a better understanding of the ways in which cooperation influences future interactions. This is an important issue to be explored because such discussions may allow the reconciliation of whether there is a genetic disposition to behave in a cooperative and altruistic manner (e.g., Warneken, 2016) based on personal experiences among individuals. Differences in ontogeny that are caused by cultural aspects may be related to the notion of cultural learning (e.g., Tomasello, 2016). An essential aspect of cultural learning is related to learning about others behavior.

## Data Availability Statement

The raw data supporting the conclusions of this article will be made available by the authors, without undue reservation.

## Ethics Statement

The studies involving human participants were reviewed and approved by Ethics committee of the University of São Paulo (CAAE protocol: 64336716.4.0000.5561). The patients/participants provided their written informed consent to participate in this study.

## Author Contributions

CS and MB conceived the study. CS designed the experiment and collected the data. CS, KC, MB, JA-R, IS, and KL-D wrote the manuscript. JS carried out the statistical analysis. CS and KC drew the tables and the figures. All authors contributed to the article and approved the submitted version.

## Conflict of Interest

The authors declare that the research was conducted in the absence of any commercial or financial relationships that could be construed as a potential conflict of interest.

## Abbreviations

AI: Advantageous Inequity
DI: Disadvantageous Inequity
CI: Cooperative Interaction
UI: Uncooperative Interaction.
